# Exploring motivations, information behavior, perceptions, and intentions among dietary supplement users: a cross-sectional survey study in Germany

**DOI:** 10.3389/fnut.2025.1663562

**Published:** 2025-10-07

**Authors:** Robin Janzik, Johanna Geppert, Patricia Müller, Inka Notz, Henri Obstfeld, Bianca Roth, Anna-Maria Volpers, Gaby-Fleur Böl

**Affiliations:** Department Risk Communication, German Federal Institute for Risk Assessment (BfR), Berlin, Germany

**Keywords:** dietary supplements, motivation, information behavior, risk perception, benefit perception, future use intention, survey study

## Abstract

**Objective:**

The use of vitamins, minerals, or botanicals via dietary supplements (DS) is increasing in the general population despite unclear benefits and the potential risks they pose to otherwise healthy individuals. A number of studies have made attempts to explain past use based on isolated individual (e.g., age), motivational (e.g., maintenance of health), informational (e.g., labeling), or perceptual (e.g., risks and benefits) variables. However, little research has examined explaining factors comprehensively among users, or explored future intentions to expand use beyond one’s current consumption.

**Methods:**

This study aimed to address these gaps by analyzing nationally representative survey data from Germany (*N* = 1,071). Participants were quota-sampled based on gender and age groups, educational levels, and federal states. Identifying DS users was based on the self-reported intake of 61 different substances, while measurements included items on health-related characteristics as well as DS-related motivations, information behavior, perceptions, attitudes, and intentions.

**Results:**

Consistent with prior research, DS users (76.9%, *n* = 824) tended to be female, younger, more health-conscious, and health-literate compared to non-users. Analysis of user data suggested five distinct motivational factors: preventive, social, vulnerable, unhealthful, and situational. Users reported to receive information about DS from different sources only rarely and to feel only moderately informed. Further, users’ perceived risk and benefit of using DS were inversely related and associated with their general attitude toward the substances. Intentions to expand use were predicted by younger age, preventive as well as social motivations, and benefit perceptions.

**Conclusion:**

These results indicate that both past and future DS use is associated with diverse reasoning and own, primarily positive, judgements, potentially rooted in a confounding of perceived risk and benefit. Science communicators may build on these results by considering the conditions in which decisions for DS use are made.

## 1 Introduction

Dietary supplements (DS) are a collective term for various substances that are intended to positively complement regular food intake. In several countries, such as Germany, their ingredients are not allowed to be pharmacologically active. Thus, DS are regulated as food and, unlike medical products, not subject to strict approval and quality control. DS are divided into several main categories, including vitamins, minerals, and botanicals. Vitamins, as organic compounds, are essential for the human metabolism but cannot be entirely produced by the body. For example, vitamin C, supports the immune function (1). Minerals, as inorganic food components, are divided into macro or bulk elements (e.g., sodium) and micro or trace elements (e.g., iron). Magnesium, for example, is used to prevent cramps or enhance athletic performance (2). Botanicals comprise herbal ingredients or extracts. One example is ashwagandha powder, which may reduce stress or improve sleep (3). DS also include amino acids [e.g., branched-chain amino acids for muscle building; (4)], hormones [e.g., melatonin for sleep disorders; (5)], or fatty acids [e.g., omega-3 for heart health; (6)].

However, using DS has also been associated with potential health risks. In otherwise healthy individuals who adhere to a balanced diet, it is generally expected that adequate levels of vitamins and minerals are met through dietary sources alone. While an oversupply of these nutrients through food intake is not to be assumed, it can occur with the intake of high-dose DS [e.g., (7)]. Too high concentrations of fat-soluble vitamins such as vitamin A, D, or E may be associated with liver, heart, or kidney problems (8, 9). A similar association has been observed with excessive mineral intake, with studies indicating that too much iron can be related to the risk of short-term gastrointestinal problems (10). The expected positive effect of the botanical ashwagandha on sleep may be obscured by possible constipation (11). It is important to note the significant diversity among the described substances, both in terms of potential benefits and risks. Therefore, it is necessary to consider them as separate entities, and thus, assess individual exposure to DS in a nuanced manner.

Although the complex situation of balancing benefits and risks should require individuals to carefully consider taking DS, studies have been pointing to a high use for years (12). Results from surveys on the rates of users in different countries vary greatly. In a study in Germany by Kurzenhäuser-Carstens et al. (13), 57% of participants reported having purchased DS for personal use in the last 12 months. Studies from the USA have indicated different rates of between around 34% (14) and 49% (15) for a similar time period. In Denmark, the rate was higher at 71% users (16) than in the Netherlands at around 53% (17) and in France at 41% (18). More recent studies have shown slightly lower user rates of around 39% in Japan (19) and around 54% in China (20). By comparison, studies using student samples often report higher rates of users above 50% (21–23). However, it is important to note that users were defined very differently and studies rarely covered the entire range of DS [see also (24, 25)].

Against this background, the question which factors are associated with the use of DS becomes pertinent. Prior research suggests that they can be categorized into four main areas. First, previous studies have pointed to the importance of the individual background, comprising sociodemographic characteristics, such as education [e.g., (16)], and health-related variables, such as health consciousness, suggesting those concerned about their physical well-being may be more likely to resort to DS to stay healthy or even to prevent potential health problems [e.g., (26)]. Second, studies have examined different motivations for use and, for example, indicated that the need for health management is related to the use of DS (27). Third, more recently, research has focused on information behavior. In addition to interpersonal sources, such as physicians, friends, and family, as starting points for information, DS are discussed and advertised on traditional media, such as TV, magazines, and radio (28). In recent years, digital and social media have become more important (29), leading researchers to also explore the role of influencers, for example from the sports sector, as opinion leaders promoting use, sometimes with undue health promises [(30, 31); see also (32)]. Moreover, the labeling of products and presentation of information could be relevant to consumers’ decision-making [e.g., (33)]. And fourth, another strain of studies has looked at the role of perceptions and attitudes, for example, suggesting that a positive attitude toward substances is associated with DS use [e.g., (17)].

To better understand the increasing use of DS, it also seems to be fruitful to examine intake-related intentions, reflecting the likelihood of future behavior (34). However, intentions have received little attention in research on DS. An exploratory study by Yang et al. (35) demonstrated that the perceived importance of health was positively associated with intentions to use DS in the future. Study results also indicate that intention to use becomes less likely with higher knowledge about the substances (20). What has been neglected in the literature so far is the idea of expanding the current use of DS. Investigating corresponding intentions seems interesting in view of the increasing popularity of emerging DS (e.g., aloe) beyond more established ones (e.g., vitamin D), as users seem willing to test the potential effects of other available products.

Therefore, this study has twofold aims: first, to describe individual differences in DS users and non-users, and to analyze motivations, information behavior, and perceptions among those who have reported taking them in the past; and second, to take a more in-depth look at factors which explain intentions to take more DS in the future. To investigate these aspects, we draw on data from a representative online survey in Germany.

## 2 Literature review on factors of DS use

### 2.1 Individual factors linked to DS use

In previous research, various individual factors for DS intake have been investigated, such as demographics and health-related variables, including lifestyle or dietary habits.

Most studies show that women are more likely to use DS compared to men [e.g., (15, 16, 36); see also (24)]. In terms of age, earlier studies have shown that older participants were more likely to be users [e.g., (13, 16); see also (24)], while more recent studies have indicated an inverse relationship [e.g., (36, 37)] or little difference (17). Those with a higher level of education may be more aware of the nutritional gaps that DS can fill and, as a result, be more likely to incorporate the substances in their diet [e.g., (16)]. Indeed, DS use appears to be more widespread among individuals with higher education [e.g., (16, 38)].

Concerning lifestyle-related factors, prior research has demonstrated that those already in good health condition [e.g., (36, 39); see also (24)] and with more individual sports practice (40) tend to have a higher DS intake. However, few studies have further explored these links, potentially uncovering underlying mechanisms behind effects.

More recently, studies have begun to investigate health-related individual factors such as health consciousness. Health consciousness is defined as the tendency to seek to understand and potentially control influences on one’s own health [e.g., (41)]. Therefore, a link to DS use seems plausible. Royne et al. (26) showed that health consciousness was positively associated with attitudes toward DS, while these attitudes, in turn, had a positive effect on perceived benefits and a negative effect on perceived risks. Other important variables in health decisions are considered to be types of literacy. Literacy in this context is defined as the understanding of, access to, and individual ability to assess health-related information [e.g., (42)]. With regard to DS, so far, few studies have looked at the role of this variable. As a first exploration, Yang et al. (35) examined media literacy among students, conceptualizing it as an understanding of authors and audiences, messages and meanings, as well as representation and reality. Their results suggested a negative link between media literacy and current DS use.

### 2.2 Motivations driving DS use

Studies aimed to capture the diversity of different motivations for DS use, often also using terms such as reasons or motives. For example, in an early qualitative study, Nichter and Thompson (27) found that overall use was based on pragmatic, strategic, and ideological reasons. The authors identified 30 different motivations, which they categorized into the areas of health management (e.g., healthy aging), resisting illness (e.g., boosting immune system), illness management (e.g., slow progress of disease), ideology (e.g., personal freedom), and harm reduction (e.g., stress relief). Bailey et al. (15) followed a similar approach in a quantitative survey by presenting respondents with 22 potential reasons for DS intake. Among all adults, the highest level of agreement was found for using DS to generally improve and maintain health, while specific areas (e.g., bone health or eye health) were reported less frequently. Other studies took a more concise approach with regard to the number of possible motivations. Barnes et al. (43), for example, presented respondents with six reasons per DS, among them also a socially oriented one (i.e., “I was told to do so”). Moreover, they included a few specific ones (e.g., bone health for calcium). Their results again point to health as the primary motivation for DS intake.

Thus, existing studies have predominantly focused on health aspects for motivational factors. The potential role of other motivations such as lifestyle choices, general health trends, or socially oriented reasons (e.g., recommendations) have been less investigated to date. The latter seem to be particularly important as DS are not only recommended by interpersonal sources (e.g., physicians) but also by influencers on social media, who have been shown to be actively advertising and providing information on DS (30, 31). In addition, there have been few attempts to identify overarching motivations that would facilitate the identification of different types of DS users.

### 2.3 Role of information sources and presentation for DS perceptions

A number of studies report findings on the extent to which information about DS and related information behavior affect the perception and use of the substances in question. Experimental approaches are often applied to investigate the impact of certain elements of the presentation of information on intake-related variables (44–46). For example, Mason et al. (45) showed that government-mandated disclaimers did not affect efficacy and safety perceptions of DS. However, a warning (compared to a disclaimer) did lead to lower perceived efficacy and safety. Further research has indicated effects of the ways information on DS are presented on consumers’ assessments (33, 47, 48). A comparative study conducted by Aschemann-Witzel and Grunert (47) in Denmark and the US showed that non-scientifically framed information influences individuals’ assessments of the effect of a DS in a positive sense. However, this was only observed in Denmark; in the US, scientifically framed information was more likely to lead to a positive assessment.

Beyond this exploration of elements of information that DS users may not come across themselves, few studies have examined the role of media use and preferences for information sources for DS intake. In an early study by Okleshen Peters et al. (49), participants reported that they were most likely to receive information about vitamins from doctors compared to parents, friends, the media in general, and pharmacists. Pajor et al. (17) showed that social support, operationalized by the extent of recommendations from the social environment, had a positive effect on the use of DS. Furthermore, specific media use, such as fitness content, has been found to promote the use of certain DS [e.g., amino acids; (50)]. However, as Wang et al. (51) summarized, research to date has focused more on business-to-consumer communication and its credibility than on effects on actual DS intake.

In summary, existing study results suggest that the perception of DS may not only be associated with the presentation of certain information but also with receiving information from certain sources. This also raises the question of the extent to which the reception of information is linked to respondents’ knowledge of DS. A focus on certain sources could be associated with a more pronounced perception of knowledge or feeling of being informed. Karbownik et al. (52) conducted an initial study on this in the context of DS, focusing the effect of advertising. However, given the effect of social support, the role of other, particularly interpersonal sources for the development of knowledge has been less investigated.

### 2.4 Perceptions and attitudes associated with DS use

Research suggests that perceptions, for instance perceived risk and benefit, as well as attitudes have an impact on health-related behavior (53, 54). Studies on various risks have shown that the perception of risk and benefit is often negatively correlated. This raises the question of whether this correctly reflects reality (higher risk corresponds to lower benefit) or whether these two aspects cannot be assessed independently of each other (there are benefits, but also risks). Alhakami and Slovic (55) summarized that this negative correlation, combined with a link to general attitudes, suggests a confounding of risk and benefit, indicating that people are not adequately able to assess the two dimensions separately. In the context of DS, where both dimensions of each substance are equally challenging to assess for laypersons, there is a paucity of research investigating this issue.

Nevertheless, prior studies have looked at perceptions and attitudes toward DS. In a study of non-users of vitamin supplements in Australia, O’Connor and White (56) showed that attitude and subjective norms were predictors of willingness to use vitamins. Pajor et al. (17) also found this positive association between attitude and DS use and demonstrated that use became more likely with higher risk perception. Further studies demonstrated links between knowledge perceptions and DS use (20) as well as attitudes toward the products (57).

Overall, given the paucity of studies that have examined perceptions and attitudes in conjunction with the other factors outlined, further investigation appears warranted. A significant research gap pertains to the relationship between perceived risk and benefit in relation to DS.

### 2.5 Aims of the study

In light of the outlined gaps in the existing social science research on DS, the current study had two aims. First, we aimed to provide a comprehensive analysis of the characteristics, motivations, information behavior and perceptions of DS users, adopting a broad approach with a multitude of substances. Secondly, we examined which factors predict intentions to expand own DS use in the future.

To this end, we drew on a comprehensive dataset from a population-representative survey on this topic conducted in 2024. The results allow current insights into the patterns of DS use and provide evidence on variables that have received little attention to date. Considering the importance of both interpersonal sources (49) and digital platforms as well as a high presence in social media (29, 31), current data appear relevant as the information environment in which DS are discussed and advertised is dynamic. These results also provide starting points for communicators with the aim of better understanding the perceptions and behavior of DS users for tailored information services.

A first step involved exploring whether DS users differed from non-users:

RQ1: Do DS users differ from non-users in terms of demographic and general health-related characteristics?

Next, we investigated DS users’ motivations, information behavior, and perceptions:

RQ2: Which motivations are relevant among DS users?

RQ3: How do DS users receive information and how does this relate to feeling informed?

RQ4: Do DS users separate benefits and risks in their perceptions?

Lastly, we analyzed which factors are linked to intentions to use further DS in the future:

RQ5: Which health-related, motivational, informational, and perceptual factors contribute to intentions to expand DS use?

## 3 Materials and methods

### 3.1 Procedure and participants

The current research was part of a comprehensive online survey study on DS that also included aspects of use not covered in this paper.^1^ Data were collected in cooperation with a professional service provider for market and social research during September 2024. Since our study primarily had exploratory objectives, we decided not to conduct a power analysis in advance and instead aimed for a sample size of approximately 1,000 participants in order to achieve a balance between informative value and resources. Online access panels were used to recruit participants and random quotas based on combined gender-age groups, educational levels, and federal states increased the data’s representativeness.

A total of 1,156 participants completed the questionnaire after consenting to take part in a survey about nutrition and health. The service provider screened the data in terms of fast completion times (60% faster or more than the median completion time), straight-lining (answers in a 26-item scale with *SD* = 0), and implausible answers in open-ended questions. This process led to the exclusion of 85 participants, resulting in a final sample of *N* = 1,071. The mean age was 49.0 years (*SD* = 17.1), gender categories (51.1% female) and education levels (39.1% higher education) were equally distributed (see Section 4.1 for full information).

### 3.2 Measurements

#### 3.2.1 DS use

To measure *DS use*, we adopted a broad approach using four self-developed questions addressing different kinds of DS. Following a general question (“Please indicate which of the following [vitamins | minerals | botanicals | other substances] you have taken in the previous 12 months via dietary supplements (e.g., as capsules or powder)”), participants were asked to select whether they had used a total of 14 vitamins (e.g., vitamin D, beta-carotene), 17 minerals (e.g., magnesium, zinc), 12 botanicals (e.g., ashwagandha, curcumin), and 18 other substances (e.g., omega fatty acids, creatine), respectively, in the past (see Supplementary Table S1). In each of the four questions, participants could select multiple answers and name additional DS not presented. Exclusive answer options (e.g., “I have not taken any of these”) allowed to indicate that none of the listed substances were used. To be identified as users, participants were required to report having taken at least one of the presented 61 substances via DS within the previous 12 months.

#### 3.2.2 Health-related characteristics

The measurement of *health consciousness* was based on an adapted and shortened version of the Health Consciousness Scale in German (HCS-G) by Marsall et al. (58). Three items (e.g., “I’m constantly examining my health”) on a scale from 1 = “Strongly disagree” to 5 = “Strongly agree” were used to assess participants’ concerns about health and their day-to-day occurrence. Internal consistency of the mean index was satisfactory (α = 0.85, *M* = 3.7, *SD* = 0.9).

Participants’ *health literacy* was measured using the German revised eHealth Literacy Scale (GR-eHEALS) by Marsall et al. (59). We chose a measure of digital health literacy to account for DS’ presence in digital information environments (e.g., social media) in recent years. The scale comprises two factors: information seeking reflects knowledge about digital sources of relevant health information (e.g., “I know how to find helpful health resources on the Internet”), while information appraisal reflects abilities to distinguish relevant from irrelevant information (e.g., “I can tell high-quality from low-quality health resources on the Internet”). Each factor was measured using four items on a 5-point scale ranging from 1 = “Strongly disagree” to 5 = “Strongly agree.” Mean indices for both factors showed satisfactory internal consistency (information seeking: α = 0.88, *M* = 3.6, *SD* = 0.9; information appraisal: α = 0.81, *M* = 3.6, *SD* = 0.8).

#### 3.2.3 DS use motivations

*DS use motivations* were assessed by a total of 26 self-developed items. Introduced by a general question (“For which reasons do you personally take dietary supplements? I take dietary supplements because…”), these items were based on theoretical considerations in terms of intake and covered areas such as prevention (e.g., “I want to prevent illnesses or health complaints”), deficits (e.g., “I know that I am deficient in certain nutrients (e.g., according to laboratory tests)”), optimization (e.g., “I want to improve my physical or mental performance”), or the social environment (e.g., “I was personally advised to do so by a person from my private environment”). Each item was measured on a 5-point scale ranging from 1 = “Strongly disagree” to 5 = “Strongly agree.” Item statistics and further analyses are reported below.

#### 3.2.4 DS information behavior

We measured participants’ *frequency of receiving information about DS* using six items covering different interpersonal sources. These included (1) physicians, (2) pharmacists, (3) sales staff in supermarkets or drugstores, (4) partner or relatives, (5) friends or acquaintances, and, more generally, (6) people from the sports sector. Each item was measured on a 5-point scale ranging from 1 = “Never” to 5 = “Very often.”

The extent to which participants *feel informed about DS* was measured using five self-developed items. Based on a 5-point scale ranging from 1 = “Very bad” to 5 = “Very good,” these items covered outcomes (e.g., “Health benefits of taking dietary supplements”), intake (e.g., “Recommended maximum doses for the intake of dietary supplements”), or the legal framework (e.g., “Legal regulations and control of dietary supplements”). Calculating a sum index, higher scores corresponded to feeling more informed (range: 5–25; *M* = 14.9, *SD* = 4.6).

#### 3.2.5 DS perceptions and attitudes

*Risk and benefit perceptions* regarding the self-administered intake of over-the-counter DS (without prior consultation with a doctor) were measured using two self-developed items, respectively. On a 5-point scale ranging from 1 = “Very low” to 5 = “Very high,” participants were asked to rate the health risk (*M* = 2.9, *SD* = 0.9) and health benefit (*M* = 2.9, *SD* = 0.9; see Supplementary Table S2 for item distributions).

The measurement of the *general attitude toward DS* comprised two self-developed items. Participants were asked to rate both a potential positive impact (“Taking dietary supplements also has a positive impact on healthy people with a balanced diet”) and the absence of a positive impact (“For healthy people with a balanced diet, dietary supplements are not necessary for the supply of nutrients”) on a 5-point scale ranging from 1 = “Strongly disagree” to 5 = “Strongly agree.” After recoding the negatively-worded item, a mean index with acceptable internal consistency (despite being a two-item measure; α = 0.62) was built (*M* = 2.7, *SD* = 0.9).

#### 3.2.6 Intentions to expand DS use

One self-developed item measured *intentions to expand DS use* in the future. Participants were asked to assess their likelihood taking DS that they had not previously taken in the next 12 months. Based on a 5-point scale ranging from 1 = “Very unlikely” to 5 = “Very likely,” the overall mean was comparatively low (*M* = 2.4, *SD* = 1.3; see Supplementary Table S2 for item distribution).

### 3.3 Data analysis

Answering RQ1 involved calculating the rate of DS users and conducting χ^2^- and *t*-tests for comparisons with non-users; *p*-values were adjusted for multiple comparisons based on the Holm method. For RQ2, we investigated motivation items using exploratory factor analysis after inspecting distributions and checking the data for suitability based on Kaiser–Meyer–Olkin (KMO) and Bartlett’s test (60). Information behavior (RQ3) was explored by inspecting means for sources of information on DS and correlations with feeling informed. Associations between risk and benefit perceptions and attitudes (RQ4) were also explored by computing correlations. Finally, for RQ5, a hierarchical regression analysis was conducted to predict intentions to expand DS use. Predictors were included step-wise to investigate the extent to which the different sets of variables added to explaining variance. Inspecting variance inflation factors of all models did not show signs of multicollinearity issues.

We performed all analyses in *R* [version 4.4.1; (61)].

## 4 Results

### 4.1 Differences between users and non-users (RQ1)

Before investigating differences regarding demographic and general health-related characteristics between users and non-users of DS (RQ1), we identified a rate of 76.9% (*n* = 824) users in the full sample. Table 1 shows that DS users, overall, did not differ significantly from the full sample in terms of gender, age, and education level distributions.

**Table 1:** Participants’ demographics for the full sample and DS users.

	Full sample (*n* = 1,071)		DS users (*n* = 824)		χ^2^	*p*
	*n*	%	*n*	%		
Gender						
Male	517	48.3%	374	45.4%	1.60	0.448
Female	547	51.1%	445	54.0%		
Non-binary	7	0.7%	5	0.6%		
Age						
16–19 years	43	4.0%	34	4.1%	2.36	0.937
20–29 years	138	12.9%	113	13.7%		
30–39 years	173	16.2%	144	17.5%		
40–49 years	184	17.2%	148	18.0%		
50–59 years	219	20.4%	162	19.7%		
60–69 years	180	16.8%	131	15.9%		
70–79 years	94	8.8%	68	8.3%		
80 + years	40	3.7%	24	2.9%		
*M*, *SD*	49.0	17.1	47.9	16.8		
Education						
Lower	269	25.1%	191	23.2%	2.08	0.556
Medium	372	34.7%	292	35.4%		
Higher	419	39.1%	336	40.8%		
Other	11	1.0%	5	0.6%		

*N* = 1,071.

To contextualize the rate of DS users, Table 2 shows that minerals were the most used substance group, followed by vitamins, while other DS and botanicals were comparatively less used by participants. Looking at the top-ten individual DS used, magnesium was used by more than half of participants. This was followed by several vitamins (D, B12, C), with well-known minerals such as zinc, calcium, and iron also having been used by more than a fifth of participants.

**Table 2:** Rate of DS users in the full sample by substance group and top-ten individual substances.

	Rate	
	*n*	%
Substance group		
1. Minerals	692	64.6%
2. Vitamins	647	60.4%
3. Other	431	40.2%
4. Botanicals	293	27.4%
Top-ten individual substances		
1. Magnesium	584	54.5%
2. Vitamin D	431	40.2%
3. Vitamin B12	364	34.0%
4. Vitamin C	340	31.7%
5. Zinc	280	26.1%
6. Calcium	261	24.4%
7. Iron	243	22.7%
8. Folic acid (vitamin B9)	193	18.0%
9. Vitamin B6	191	17.8%
10. Omega fatty acids	189	17.6%

*N* = 1,071. Substance group and top-ten individual substances were arranged from highest to lowest *n*.

Concerning demographic differences between users and non-users of DS, Table 3 shows significant differences regarding gender and age. Users were found to be more likely to be female and younger. However, users were not significantly more likely to be higher educated compared to non-users, although the difference almost reached statistical significance (*p* = 0.078).

**Table 3:** Demographic and health-related differences between DS users and non-users.

	Users (*n* = 814)	Non-users (*n* = 239)	χ^2^/*t*	*p*	*p*_adj._	φ/*d*
	%/*M* (*SD*)	%/*M* (*SD*)				
Demographics						
Gender (female)	54.2%	42.3%	10.03	0.002	0.003	0.10
Age (years)	48.0 (16.8)	52.5 (17.4)	3.57	< 0.001	0.001	0.27
Education (higher)	40.9%	34.3%	3.10	0.078	0.078	0.05
Health-related characteristics						
Health consciousness	3.8 (0.8)	3.5 (0.8)	−4.96	< 0.001	< 0.001	0.40
Health literacy – Seeking	3.6 (0.8)	3.3 (0.8)	−4.96	< 0.001	< 0.001	0.38
Health literacy – Appraisal	3.7 (0.8)	3.4 (0.8)	−4.84	< 0.001	< 0.001	0.37

*N* = 1,053. Due to small cell sizes, non-binary participants (*n* = 7) and those not to be categorized into lower, medium, or higher education (*n* = 11) were excluded from analysis. Education was dummy-coded into lower/medium (0) vs. higher education (1).

Further, DS users differed significantly from non-users with regard to all three investigated indicators of health-related characteristics. Users were more likely to be health-conscious and health-literate in terms of knowledge of sources for health information (information seeking) and competence to assess them (information appraisal). Differences were of comparable magnitude across the three constructs (*d* = 0.37–0.40).

### 4.2 Dimensionality of use motivations (RQ2)

**Figure 1:**
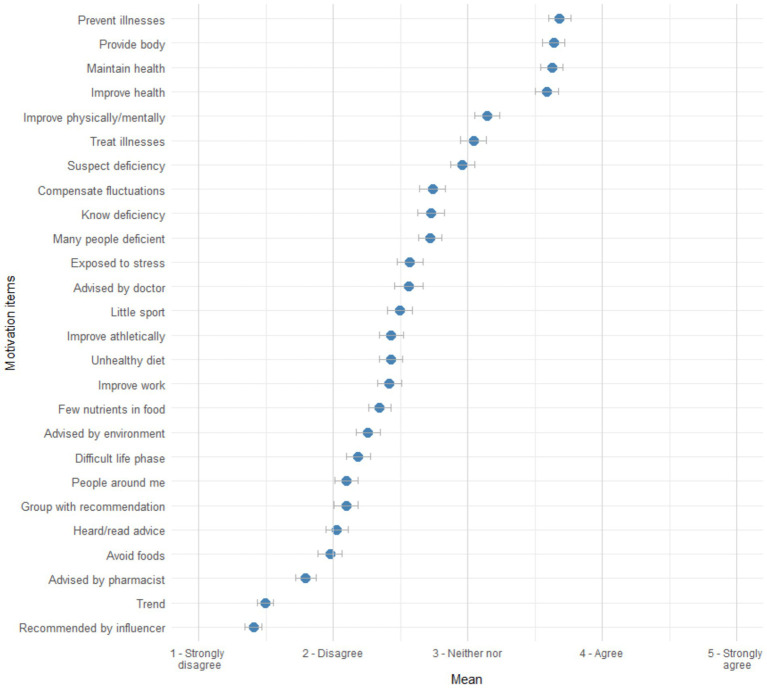
Distribution of DS use motivation items. *n* = 824. Items were arranged from highest to lowest mean. Error bars represent 95% CIs.

To examine relevant motivations among DS users (RQ2), we first inspected means of the 26 measured motivation items. Figure 1 shows that, on a descriptive level, mostly general, prevention- and curation-related motivations were important. Highest mean values were observed for taking DS to prevent illnesses, provide the body with nutrients, maintain health, and to improve health. By comparison, mean values for items addressing the social environment, such as taking DS because of trends or recommendations from social media influencers, were considerably lower. Overall, items arranged around the middle of the scale, with mean values not exceeding 4.

To explore the dimensionality of the motivation items, we used principal component analysis (*n* = 824, KMO = 0.89, Bartlett’s test: χ^2^ (325) = 7572.38, *p* < 0.001). Visually inspecting the scree-plot and parallel analysis (50 iterations) suggested a five-factor solution (see Table 4). We removed four items given low primary loadings (< 0.50) and another three items because of high cross-loadings [> 0.40; (60)]. Five items comprised the first factor reflecting a *preventive motivation* for taking DS to guard oneself against potential health problems. The second factor reflected a *social motivation* including five items addressing intake based on information received from others. Three items loaded onto the third factor representing the motivation to take DS due to knowledge about one’s own vulnerability and corresponding advice from experts (i.e., *vulnerable motivation*). A fourth factor comprising three items reflected the motivation to take DS to balance out unhealthful lifestyle choices (i.e., *unhealthful motivation*). Lastly, the fifth factor for a *situational motivation* (three items) combined appraisals of the use of DS in specific circumstances, such as periods of stress at work or in personal life.

**Table 4:** Summary of principal component analysis for DS use motivations.

	Preventive	Social	Vulnerable	Unhealthful	Situational
Factor loadings					
Prevent illnesses	0.76				
Maintain health	0.74				
Provide body	0.72				
Improve health	0.61				
Many people deficient	0.50				
Trend		0.70			
People around me		0.70			
Recommended by influencer		0.66			
Heard/read advice		0.65			
Advised by environment		0.63			
Advised by doctor			0.80		
Group with recommendation			0.73		
Know deficiency			0.60		
Unhealthy diet				0.78	
Little sport				0.72	
Few nutrients in food				0.53	
Improve work					0.75
Improve athletically					0.70
Exposed to stress					0.64
Factor summary					
Eigenvalue	3.52	2.96	2.60	2.55	2.48
Variance explained	25%	21%	18%	18%	18%
Number of items	5	5	3	3	3
*M*	3.5	1.9	2.5	2.4	2.5
*SD*	0.9	0.8	1.1	1.0	1.1
α	0.79	0.76	0.71	0.70	0.72

*n* = 824. Factor loadings < 0.40 are not shown for clarity. Means, standard deviations, and Cronbach’s α for each factor were based on the final number of items. Items not considered in corresponding factor due to low primary loadings < 0.50: “Compensate fluctuations,” “Suspect deficiency,” “Treat illnesses,” “Avoid foods.” Items not considered in corresponding factor due to high cross-loadings > 0.40: “Advised by pharmacist,” “Improve physically/mentally,” “Difficult life phase”.

All five factors showed satisfactory internal consistency (α = 0.70–0.79; see Table 4). For further analysis (RQ5), we calculated mean indices for each motivational factor, respectively.

### 4.3 Relationship of receiving information and feeling informed (RQ3)

Exploring DS users’ information behavior (RQ3), we found that on average participants received information from the investigated interpersonal sources only rarely. Friends and acquaintances were identified as the primary source of information (*M* = 2.23, *SD* = 1.0), followed by partners or relatives (*M* = 2.22, *SD* = 1.1), and physicians (*M* = 2.19, *SD* = 1.1). The mean value for pharmacists as an information source were comparatively lower (*M* = 1.96, *SD* = 1.0), while values for people from the sports sector (*M* = 1.77, *SD* = 1.0) and sales staff in supermarkets and drugstores ranked lowest (*M* = 1.43, *SD* = 0.8). Overall, there were no meaningful differences between sources on a descriptive level, albeit with a tendency toward the closer social environment.

Further, we calculated correlations of frequencies with which users reported to receive information from the different sources and their feeling of being informed about DS (see Table 5). Feeling informed was highest positively related to the frequency of receiving information from physicians (*r* = 0.19) and pharmacists (*r* = 0.16). Overall, receiving information from neither source was negatively correlated with the sum index of feeling informed.

**Table 5:** Intercorrelations of frequency of information regarding DS from different sources and feeling informed.

Variables	1	2	3	4	5	6	7
(1) Source – Physicians	–						
(2) Source – Pharmacists	0.54***	–					
(3) Source – Sales staff in supermarkets or drugstores	0.22***	0.40***	–				
(4) Source – Partner or relatives	0.21***	0.24***	0.29***	–			
(5) Source – Friends or acquaintances	0.20***	0.30***	0.36***	0.49***	–		
(6) Source – People from the sports sector	0.23***	0.31***	0.42***	0.31***	0.36***	–	
(7) Feeling informed	0.19***	0.16***	0.09*	0.10**	0.08*	0.09*	–

*n* = 824. *p < 0.05, **p < 0.01, ***p < 0.001. Pearson correlations based on list-wise deletion.

### 4.4 Associations between risk and benefit perceptions and attitude (RQ4)

To answer the question whether there was an inverse relationship between DS users’ risk and benefit perceptions (RQ4), we first found similar distributions of the two variables, with a significant difference in means of 0.29, 95% CI [0.19, 0.39]. A higher mean of perceived benefit (*M* = 3.14, 95% CI [3.07, 3.20]) was observed compared to perceived risk (*M* = 2.84, 95% CI [2.78, 2.91]), *t* (802) = −5.55, *p* < 0.001.

Table 6 shows that perceptions of risk and benefit of DS were negatively correlated. Further, we found perceived risk to be significantly negatively and perceived benefit to be significantly positively correlated to overall evaluations of DS, as measured by the index for general attitude. The correlation coefficient for benefit perception and attitude was higher by comparison. Consequently, individuals consuming DS and perceiving them as beneficial were less likely to perceive risks and more likely to hold a favorable attitude toward them. In contrast, higher risk perceptions were associated with a more unfavorable attitude toward DS.

**Table 6:** Intercorrelations of risk and benefit perceptions and general attitude regarding DS use.

Variables	*M* (*SD*)	1	2	3
(1) Risk perception	2.8 (0.9)	–		
(2) Benefit perception	3.1 (0.9)	−0.31***	–	
(3) General attitude	2.8 (0.9)	−0.18***	0.41***	–

*n* = 824. *p < 0.05, **p < 0.01, ***p < 0.001. Pearson correlations based on list-wise deletion.

### 4.5 Factors predicting intentions to expand use (RQ5)

To investigate RQ5, we conducted a hierarchical regression predicting intentions to expand use among those already taking DS. The first step consisted of demographics as controls, with the second step including variables for health-related characteristics. The third and fourth step involved the entry of motivation factors and information behavior (feeling informed). In the final, fifth step, perceptions and attitudes were entered. All regression steps were significant (*p* < 0.01; see Table 7).

**Table 7:** Hierarchical regression model predicting intentions to expand DS use.

Step	Predictor	*B*	95% CI (*B*)	*SE* (*B*)	β	*p*	*R* ^2^	Δ*R*^2^
Step 1: Demographics	Gender (1 = female)	0.02	[−0.16, 0.19]	0.09	0.01	0.864		
	Age	−0.01	[−0.01, −0.00]	0.00	−0.09	0.025		
	Education (1 = higher)	−0.02	[−0.21, 0.17]	0.10	−0.01	0.830	0.02**	–
Step 2: Health-related characteristics	Health consciousness	0.02	[−0.10, 0.14]	0.06	0.01	0.773		
	Health literacy – Seeking	0.14	[−0.05, 0.32]	0.09	0.09	0.148		
	Health literacy – Appraisal	−0.06	[−0.26, 0.14]	0.10	−0.04	0.534	0.04***	0.02**
Step 3: Motivations	Preventive	0.19	[0.06, 0.33]	0.07	0.13	0.005		
	Social	0.17	[0.04, 0.29]	0.06	0.11	0.011		
	Vulnerable	0.03	[−0.05, 0.12]	0.04	0.03	0.446		
	Unhealthful	0.06	[−0.05, 0.17]	0.05	0.05	0.269		
	Situational	0.00	[−0.10, 0.11]	0.05	0.00	0.925	0.13***	0.09***
Step 4: Information behavior	Feeling informed	0.01	[−0.01, 0.03]	0.01	0.03	0.425	0.13***	0.00
Step 5: Perceptions and attitudes	Risk perception	−0.02	[−0.13, 0.08]	0.05	−0.02	0.662		
	Benefit perception	0.21	[0.10, 0.33]	0.06	0.15	0.000		
	General attitude	0.09	[−0.01, 0.20]	0.05	0.07	0.085	0.16***	0.03***

All regression steps were based on cases without missing values (*n* = 667). *p < 0.05, **p < 0.01, ***p < 0.001. Due to small cell sizes, non-binary participants (*n* = 7) and those not to be categorized into lower, medium, or higher education (*n* = 11) were excluded from analysis. Education was dummy-coded into lower/medium (0) vs. higher education (1). Shown are values from the last step of analysis including all variables.

Predicting intentions to expand DS use, including motivation factors led to the largest increment in explained variance (Δ*R*^2^ = 0.09, *p* < 0.001), while perceptions and attitudes (Δ*R*^2^ = 0.03, *p* < 0.001) as well as health-related characteristics (Δ*R*^2^ = 0.02, *p* = 0.003) also added significantly to explaining variance. The extent of feeling informed, as a key indicator of information behavior, did not incrementally explain variance (Δ*R*^2^ = 0.00, *p* = 0.210). Several variables emerged as predictors of intentions to expand the use of DS in the following months. Regarding demographics, younger age predicted intentions (β = −0.09). Further, both a preventive (β = 0.13) and a social motivation (β = 0.11) were positive predictors. The highest effect size among the significant predictors was observed for perceived benefit (β = 0.15), whereas general attitude narrowly failed to reach significance (β = 0.07, *p* = 0.085).

## 5 Discussion

The central contribution of the current study is the comprehensive investigation of the characteristics of DS users, the motivations behind their use, the sources of information they consult, and their perceptions of the substances. In addition, this study is among the first to provide insights into factors that are linked to the extent to which individuals intend to expand their own use. This goes, for instance, beyond initial analyses on individual motivations (15), the importance of physicians as an information source (49), and attitudes predicting intentions (56). In light of the sample size and its representativeness, as well as the inclusion of a large number of DS to identify users, the study also contributes to developing high-quality standards for data on which the state of research is based.

### 5.1 Understanding DS users’ motivations, information behavior, and perceptions

Exploring the demographic and health-related characteristics of DS users compared to non-users (RQ1) showed five out of six investigated differences to be significant. With DS users more likely to be female and younger, as well as tending to be rather highly educated, our current results align with the trends demonstrated in the literature (24, 25). Noteworthy, this pattern persists, indicating that the user group does not appear to become more diverse, even though access to the products broadened in recent years [e.g., (62)]. Future studies should explore which variables explain these demographic trends. Health consciousness and literacy are only starting points for this. Our results showed higher levels among users, which seems plausible in light of the assumed health benefits of DS (26, 35, 63). However, given the often unclear benefits for healthy individuals and potential risks of some substances, the question arises as to whether health consciousness or literacy should be considered an ideal orientation for decision-making regarding DS.

In terms of motivations (RQ2), our study is among the first to identify overarching motivational factors comprising various individual ways of reasoning. Unlike most prior research, which focused on isolated health-related motivations [e.g., (15, 43)], our results add a social component and consider situational intake. Interestingly, comparing means of factors, a social motivation was relatively less important, even though the potential role of trends and recommendations by influencers for DS consumption is a much-debated topic. The fact that DS use does not only occur in the context of concerns for one’s own health or due to perceived vulnerability should be taken into account in future research. In addition, future studies should continue to explore motivations by attempting to replicate the structure found here, also using confirmatory approaches.

Regarding information behavior (RQ3), our results showed that DS users only rarely received information about these products from different sources of information. Moreover, this was associated with a rather low subjective feeling of being informed about the topic. It is important to note that our measurement focused on interpersonal sources. This raises the question of how users acquire their desired information, as it is conceivable that own media usage is a more important source to gain insight [e.g., (50)]. More generally, users may rather rely on their own judgment for relevant information, being critical of the stance and knowledge of the social environment and health professionals (64). Low correlations between individuals’ perceived information levels and interpersonal information sources support this conclusion.

Results on the investigation of the relationship between perceived risk and benefit (RQ4), as originally suggested by Alhakami and Slovic (55), showed a pattern common to several risks, with both constructs showing a negative correlation and an additional association with attitudes. These associations suggest the interpretation of a potential confounding of risk and benefit (55) but they may also be rooted in affect guiding interpretation (65). One possible conclusion is that individuals understate potential risks of DS when they see benefits, but this interpretation should be further investigated in future studies. Further research should also make efforts to rule out a possible measurement artifact through semantic contrast by exploring different scales. Ultimately, perceptions of specific substances should also be looked at separately, especially as assessing the risks and benefits of DS as a whole can be challenging researchers and consumers alike.

### 5.2 Factors affecting future DS use intentions

Investigating factors associated with intentions to expand DS use among current users (RQ5) provided a deeper understanding of the role of demographic and health-related characteristics, motivational factors, information behavior, as well as perceptions and attitudes. The comparatively low mean value of intentions (*M* = 2.4) indicates that the idea of increasing the consumption of DS beyond current levels is not a particularly prevalent one. This is consistent with other survey results which also show only moderately high means of future purchase intentions related to DS [e.g., (31)]. Consequently, it can be hypothesized that some segments of the population might, thus, be content with their current choices of intake. However, this may also indicate gaps in information, as the use of additional substances without knowing their potential benefits would require a general ‘more-is-better’ stance [see also (33)].

Younger age was associated with greater intentions to expand DS use. Openness or sensation seeking could be relevant factors here as individual experience might lead to a better understanding of which DS have the desired outcomes (66). This also suggests that, in this context, demographic factors should not only be controlled for alongside more psychological factors, but also considered as potential explanatory variables.

Health-related variables also, overall, contributed significantly to predicting the likelihood of increasing DS consumption, suggesting that individuals who are already engaged in beneficial health behaviors may be more receptive to expanding their DS intake (26, 35, 63). However, none of the investigated variables individually reached significance. Therefore, this result should be interpreted with caution.

Further, results indicate that motivation factors are most relevant in explaining intentions to expand DS use. Specifically, both preventive and social motivations emerged as significant positive predictors. Individuals who perceive DS as a means of maintaining health or are driven by thoughts about their social environment promoting use were more likely to expand it. Aligning with prior research, the idea of prevention is important for individual health-related decisions [e.g., (15, 67)] and the influence of peers and societal norms contributes to this behavior as well (17, 68). The other three factors measured (vulnerable, unhealthful, situational) played a less significant role. This could be explained by these motivations being more specific (e.g., knowing a deficit and the corresponding substance) and event-related (e.g., knowing a substance to solve an acute problem).

Perceptions and attitudes were also relevant predictors of intentions, with perceived benefit emerging as the strongest influence. Advantages compared to disadvantages represented by potential risk seem to be more decisive for increasing use, while controlling for each other. Echoing this result, Bearth and Siegrist (54), in one of the few meta-analyses in this field, also found a tendency for a higher effect of benefit as opposed to risk perceptions on the acceptance of food technologies.

Overall, the results for future use intentions point to similar patterns as those observed in past use, with certain motivations and the perception of positive effects playing a role. Noteworthy, the amount of variance explained with *R*^2^ = 0.16 in the model with all the variables examined was not particularly high. Although this is not relevant for the assessment of the individual effects and still allows statements to be made about the importance of the variable blocks [e.g., (69)], the value indicates that other, external factors that have not been included here could be relevant. Economic factors such as price or product availability might influence the development of intentions as these are boundary conditions to be able to buy and use a new DS. Further, prior advice from health professionals could form how individuals assess the need for and advantages of additional substances over time.

### 5.3 Implications for science communication and public health messaging

The results of this study offer implications for science communication and public health messaging about DS. In terms of targeted information, the results showed that users of DS are more likely to be younger, female, and more health-conscious. At the same time, this provides insights into which segments of the population are more likely to be non-users who should also be provided with relevant information based on their characteristics.

Additionally, the findings suggest that different motivational factors are associated with the use of DS. For communicators, it is therefore important to understand that these motivations can vary and may be associated with specific information needs. Given that preventive and social motivations, in particular, were found to affect the expansion of DS use, they should be taken into account when designing messages. For example, for preventive users, information about long-term risks and benefits could encourage further engagement with the topic, whereas for socially motivated users, approaches that promote critical evaluation of received advice or strategies for dealing with misinformation could play an important role [see also (52)].

Given the results on information behavior, it seems prudent for communicators to place greater emphasis on reliable information sources concerning DS. With feeling informed being correlated mostly with receiving information from physicians and pharmacists, the role of healthcare professionals next to specialized information services, such as websites [e.g., (70)], should be strengthened as starting points for guidance on the variety of DS and their potential combined effects.

Lastly, based on the results on perceived risk and benefit, communicators should factor in that consumers’ general idea of DS may in some cases not be nuanced. This calls for balanced messaging addressing both aspects and disentangling that a substance might entail specific benefits but also pose certain risks depending on exposure levels.

### 5.4 Limitations

Our study is not without limitations. First, overall, we adopted an exploratory approach based on quantitative data investigating mostly correlational patterns between variables. To examine causal links, future research should integrate longitudinal approaches.

Second, our study focused on DS in general. As a very heterogeneous group of substances with different use patterns, it is challenging to evaluate it uniformly. Respondents could have based their general assessment of DS on that of a specific substance. Future studies should therefore attempt to replicate the results with regard to substance groups that are dominantly used [e.g., botanicals; see also (71)] or even individual substances [e.g., vitamin D; see also (72)].

Third, accordingly, we used a broad approach to identify users, covering a span of 12 months. It is possible that stricter criteria (e.g., 30 days) may lead to different results, both with regard to prevalence and predicting intentions among users. Future research should extend our dichotomization by, for example, examining infrequent and frequent users, taking into account users of different substance groups.

Fourth, our analysis focused on interpersonal sources. While the frequency of receiving information from these sources has been underresearched before, a full picture requires comparing frequencies with other important sources, such as social media platforms. Future research may therefore also benefit from integrating survey and content-analytical data.

Fifth, the study used some single-item measurements, such as those for risk and benefit perceptions and intentions, due to space constraints. This limits construct validity, potentially attenuating or inflating correlations because of common-method variance. In future studies, these measurements should be compared with established multi-item measurements on various constructs to improve reliability.

Finally, the results were based on a sample from Germany. Given dietary habits that may be associated with the increased use of specific DS, the results cannot be transferred with certainty to the situation in other countries or cultures. Due to the focus of previous research on Western countries, further studies on the topic in non-Western contexts would be beneficial.

## 6 Conclusion

Which motivations, information behavior, and perceptions do users of DS show and to what extent are they willing to expand their use? Based on a representative sample of the German population and using a wide range of substances to identify users, this study explored key variables to better understand past and future use of DS. Overall, the results indicate diverse (i.e., preventive, social, vulnerable, unhealthful, and situational) motivational factors, an information behavior that is less influenced by interpersonal sources, and an indistinct association in individual perceptions of risks and benefits of DS in general. Intentions to expand the range of DS taken were driven by age, motivations (preventive and social), and perceived benefits. In a time of increasing popularity of DS, the benefits of which might be unclear for otherwise healthy individuals, communicators should recognize that use is not simply determined but based on an interplay of different motivational, informational, and perceptual factors.

## Data Availability

The raw data supporting the conclusions of this article will be made available by the authors, without undue reservation.

## Footnotes

^1^These data are the basis for analyses in another journal contribution out of the scope of this article. For more information see: Obstfeld H, Lohmann M. Nahrungsergänzungsmittel im Kontext sozialer Medien: Ergebnisse einer Befragung zur Nutzung und Wahrnehmung in Deutschland [Food supplements in the context of social media: results from a survey on use and perceptions in Germany]. Bundesgesundheitsblatt, forthcoming.

## References

[1] Carr A Maggini S. Vitamin C and immune function. Nutrients. (2017) 9:1211. doi: 10.3390/nu9111211, PMID: 29099763 PMC5707683

[2] Roffe C Sills S Crome P Jones P. Randomised, cross-over, placebo controlled trial of magnesium citrate in the treatment of chronic persistent leg cramps. Med Sci Monit. (2002) 8:CR326–30 12011773

[3] Lopresti AL Smith SJ. Ashwagandha (*Withania somnifera*) for the treatment and enhancement of mental and physical conditions: a systematic review of human trials. J Herb Med. (2021) 28:100434. doi: 10.1016/j.hermed.2021.100434

[4] Sharp CPM Pearson DR. Amino acid supplements and recovery from high-intensity resistance training. J Strength Cond Res. (2010) 24:1125–30. doi: 10.1519/jsc.0b013e3181c7c655, PMID: 20300014

[5] Menczel Schrire Z Phillips CL Chapman JL Duffy SL Wong G D’Rozario AL. Safety of higher doses of melatonin in adults: a systematic review and meta-analysis. J Pineal Res. (2022) 72:e12782. doi: 10.1111/jpi.12782 34923676

[6] Elagizi A Lavie CJ Marshall K DiNicolantonio JJ O’Keefe JH Milani RV. Omega-3 polyunsaturated fatty acids and cardiovascular health: a comprehensive review. Prog Cardiovasc Dis. (2018) 61:76–85. doi: 10.1016/j.pcad.2018.03.006 29571892

[7] Rasmussen SE Andersen NL Dragsted LO Larsen JC. A safe strategy for addition of vitamins and minerals to foods. Eur J Nutr. (2006) 45:123–35. doi: 10.1007/s00394-005-0580-9, PMID: 16200467

[8] Bargagli M Ferraro PM Vittori M Lombardi G Gambaro G Somani B. Calcium and vitamin D supplementation and their association with kidney stone disease: a narrative review. Nutrients. (2021) 13:4363. doi: 10.3390/nu13124363, PMID: 34959915 PMC8707627

[9] Kaye AD Thomassen AS Mashaw SA MacDonald EM Waguespack A Hickey L. Vitamin E (α-tocopherol): emerging clinical role and adverse risks of supplementation in adults. Cureus. (2025) 17:e78679. doi: 10.7759/cureus.78679, PMID: 40065887 PMC11891505

[10] Bloor SR Schutte R Hobson AR. Oral iron supplementation—gastrointestinal side effects and the impact on the gut microbiota. Microbiol Res. (2021) 12:491–502. doi: 10.3390/microbiolres12020033

[11] Chandrasekhar K Kapoor J Anishetty S. A prospective, randomized double-blind, placebo-controlled study of safety and efficacy of a high-concentration full-spectrum extract of ashwagandha root in reducing stress and anxiety in adults. Indian J Psychol Med. (2012) 34:255–62. doi: 10.4103/0253-7176.106022, PMID: 23439798 PMC3573577

[12] Cohen PA. The supplement paradox: negligible benefits, robust consumption. JAMA. (2016) 316:1453–4. doi: 10.1001/jama.2016.14252, PMID: 27727369

[13] Kurzenhäuser-Carstens S Lohmann M Böl G-F. Zielgruppengerechte Risikokommunikation zum Thema Nahrungsergänzungsmittel [target group-specific risk communication on food supplements]. UMID Umwelt Mensch Informationsdienst. (2013):65–72.

[14] Pillitteri JL Shiffman S Rohay JM Harkins AM Burton SL Wadden TA. Use of dietary supplements for weight loss in the United States: results of a national survey. Obesity. (2008) 16:790–6. doi: 10.1038/oby.2007.136, PMID: 18239570

[15] Bailey RL Gahche JJ Miller PE Thomas PR Dwyer JT. Why US adults use dietary supplements. JAMA Intern Med. (2013) 173:355. doi: 10.1001/jamainternmed.2013.229 23381623

[16] Kofoed CLF Christensen J Dragsted LO Tjønneland A Roswall N. Determinants of dietary supplement use – healthy individuals use dietary supplements. Br J Nutr. (2015) 113:1993–2000. doi: 10.1017/s0007114515001440, PMID: 25940747

[17] Pajor EM Eggers SM Curfs KCJ Oenema A de Vries H. Why do Dutch people use dietary supplements? Exploring the role of socio-cognitive and psychosocial determinants. Appetite. (2017) 114:161–8. doi: 10.1016/j.appet.2017.03.036, PMID: 28359781

[18] Pouchieu C Andreeva VA Péneau S Kesse-Guyot E Lassale C Hercberg S. Sociodemographic, lifestyle and dietary correlates of dietary supplement use in a large sample of French adults: results from the NutriNet-santé cohort study. Br J Nutr. (2013) 110:1480–91. doi: 10.1017/s0007114513000615, PMID: 23432948

[19] Chiba T Tanemura N. Differences in the perception of dietary supplements between dietary supplement/medicine users and non-users. Nutrients. (2022) 14:4114. doi: 10.3390/nu14194114, PMID: 36235766 PMC9572052

[20] Tan DS Wang X Zhao X Zhao A. The association between the knowledge, perception, and practice of dietary supplement among Chinese adults. Front Nutr. (2024) 11:11. doi: 10.3389/fnut.2024.1493504, PMID: 39582671 PMC11583637

[21] Begdache L Kianmehr H Heaney CV. College education on dietary supplements may promote responsible use in young adults. J Diet Suppl. (2020) 17:67–80. doi: 10.1080/19390211.2018.1482983, PMID: 30252551

[22] Lieberman HR Marriott BP Williams C Judelson DA Glickman EL Geiselman PJ. Patterns of dietary supplement use among college students. Clin Nutr. (2015) 34:976–82. doi: 10.1016/j.clnu.2014.10.010 25466950

[23] Valentine AA Schumacher JR Murphy J Ma YJ. Dietary supplement use, perceptions, and associated lifestyle behaviors in undergraduate college students, student-athletes, and ROTC cadets. J Am Coll Heal. (2018) 66:87–97. doi: 10.1080/07448481.2017.1377205, PMID: 28915096

[24] Dickinson A MacKay D. Health habits and other characteristics of dietary supplement users: a review. Nutr J. (2014) 13:14. doi: 10.1186/1475-2891-13-14, PMID: 24499096 PMC3931917

[25] Papatesta EM Kanellou A Peppa E Trichopoulou A. Is dietary (food) supplement intake reported in European national nutrition surveys? Nutrients. (2023) 15:5090. doi: 10.3390/nu15245090, PMID: 38140349 PMC10871081

[26] Royne MB Fox AK Deitz GD Gibson T. The effects of health consciousness and familiarity with DTCA on perceptions of dietary supplements. J Consum Aff. (2014) 48:515–34. doi: 10.1111/joca.12051

[27] Nichter M Thompson JJ. For my wellness, not just my illness: north Americans’ use of dietary supplements. Cult Med Psychiatry. (2006) 30:175–222. doi: 10.1007/s11013-006-9016-0, PMID: 16841188

[28] Muela-Molina C Perelló-Oliver S García-Arranz A. False and misleading health-related claims in food supplements on Spanish radio: an analysis from a European regulatory framework. Public Health Nutr. (2021) 24:5156–65. doi: 10.1017/s1368980021002007, PMID: 33972003 PMC11082796

[29] Catalani V Negri A Townshend H Simonato P Prilutskaya M Tippett A. The market of sport supplement in the digital era: a netnographic analysis of perceived risks, side-effects and other safety issues. Emerg Trends Drugs Addict Health. (2021) 1:100014. doi: 10.1016/j.etdah.2021.100014

[30] Kumar N Nawaz Z Samerguy P. The power of social media fitness influencers on supplements: how they affect buyer’s purchase decision? Int J Pharm Healthc Mark. (2024) 18:27–46. doi: 10.1108/ijphm-04-2022-0037

[31] Wang EST. Effects of social media influencer credibility on their followers’ dietary supplement evaluations and purchase intentions. J Mark Commun. (2025):1–24. doi: 10.1080/13527266.2025.2462991

[32] Jungnickel K Böl G-F. Meinungsführerschaft und Risikowahrnehmung im gesundheitlichen Verbraucherschutz [Opinion leadership and risk perception in consumer health protection]. BfR-Wissenschaft (2019).

[33] Homer PM Mukherjee S. The impact of dietary supplement form and dosage on perceived efficacy. J Consum Mark. (2018) 35:228–38. doi: 10.1108/jcm-02-2017-2108

[34] Sheeran P. Intention-behavior relations: a conceptual and empirical review. Eur Rev Soc Psychol. (2002) 12:1–36. doi: 10.1080/14792772143000003

[35] Yang SC Hsu W-C Chiang C-H. The associations among individual factors, media literacy, and dietary supplement use among college students: cross-sectional study. J Med Internet Res. (2020) 22:e19056. doi: 10.2196/19056, PMID: 32865500 PMC7490677

[36] Kanellou A Papatesta EM Martimianaki G Peppa E Stratou M Trichopoulou A. Dietary supplement use in Greece: methodology and findings from the National Health and nutrition survey – HYDRIA (2013–2014). Br J Nutr. (2023) 129:2174–81. doi: 10.1017/s000711452200321x, PMID: 36210533

[37] Stoś K Woźniak A Rychlik E Ziółkowska I Głowala A Ołtarzewski M. Assessment of food supplement consumption in polish population of adults. Front Nutr. (2021) 8:8. doi: 10.3389/fnut.2021.733951, PMID: 34778335 PMC8578692

[38] Lyle BJ Mares-Perlman JA Klein BEK Klein R Greger JL. Supplement users differ from nonusers in demographic, lifestyle, dietary and health characteristics. J Nutr. (1998) 128:2355–62. doi: 10.1093/jn/128.12.2355, PMID: 9868181

[39] Dickinson A MacKay D Wong A. Consumer attitudes about the role of multivitamins and other dietary supplements: report of a survey. Nutr J. (2015) 14:66. doi: 10.1186/s12937-015-0053-9, PMID: 26134111 PMC4489202

[40] Sirico F Miressi S Castaldo C Spera R Montagnani S Di Meglio F. Habits and beliefs related to food supplements: results of a survey among Italian students of different education fields and levels. PLoS One. (2018) 13:e0191424. doi: 10.1371/journal.pone.0191424, PMID: 29351568 PMC5774790

[41] Gould SJ. Health consciousness and health behavior: the application of a new health consciousness scale. Am J Prev Med. (1990) 6:228–37. doi: 10.1016/s0749-3797(18)31009-2, PMID: 2223170

[42] Norman CD Skinner HA. eHealth literacy: essential skills for consumer health in a networked world. J Med Internet Res. (2006) 8:e9. doi: 10.2196/jmir.8.2.e9, PMID: 16867972 PMC1550701

[43] Barnes K Ball L Desbrow B Alsharairi N Ahmed F. Consumption and reasons for use of dietary supplements in an Australian university population. Nutrition. (2016) 32:524–30. doi: 10.1016/j.nut.2015.10.022, PMID: 26819063

[44] Dodge T Kaufman A. What makes consumers think dietary supplements are safe and effective? The role of disclaimers and FDA approval. Health Psychol. (2007) 26:513–7. doi: 10.1037/0278-6133.26.4.513, PMID: 17605572

[45] Mason MJ Scammon DL Fang X. The impact of warnings, disclaimers, and product experience on consumers’ perceptions of dietary supplements. J Consum Aff. (2007) 41:74–99. doi: 10.1111/j.1745-6606.2006.00069.x

[46] Dodge T Litt D Kaufman A. Influence of the dietary supplement health and education act on consumer beliefs about the safety and effectiveness of dietary supplements. J Health Commun. (2011) 16:230–44. doi: 10.1080/10810730.2010.529493, PMID: 21120738

[47] Aschemann-Witzel J Grunert KG. Influence of ‘soft’ versus ‘scientific’ health information framing and contradictory information on consumers’ health inferences and attitudes towards a food supplement. Food Qual Prefer. (2015) 42:90–9. doi: 10.1016/j.foodqual.2015.01.008

[48] Homer PM Mukherjee S. Lay theories and consumer perceptions of dietary supplements. J Consum Behav. (2019) 18:363–77. doi: 10.1002/cb.1776

[49] Okleshen Peters CL Shelton J Sharma P. An investigation of factors that influence the consumption of dietary supplements. Health Mark Q. (2003) 21:113–35. doi: 10.1300/j026v21n01_06, PMID: 15271634

[50] Frison E Vandenbosch L Eggermont S. Exposure to media predicts use of dietary supplements and anabolic-androgenic steroids among Flemish adolescent boys. Eur J Pediatr. (2013) 172:1387–92. doi: 10.1007/s00431-013-2056-x, PMID: 23748985

[51] Wang Y Neilson LC Ji S. Why and how do consumers use dietary supplements? A systematic review and thematic analysis. Health Promot Int. (2023) 38:38. doi: 10.1093/heapro/daac197, PMID: 36789498

[52] Karbownik MS Paul E Nowicka M Nowicka Z Kowalczyk RP Kowalczyk E. Knowledge about dietary supplements and trust in advertising them: development and validation of the questionnaires and preliminary results of the association between the constructs. PLoS One. (2019) 14:e0218398. doi: 10.1371/journal.pone.0218398, PMID: 31233516 PMC6590799

[53] Bearth A Cousin M-E Siegrist M. The consumer’s perception of artificial food additives: influences on acceptance, risk and benefit perceptions. Food Qual Prefer. (2014) 38:14–23. doi: 10.1016/j.foodqual.2014.05.008

[54] Bearth A Siegrist M. Are risk or benefit perceptions more important for public acceptance of innovative food technologies: a meta-analysis. Trends Food Sci Technol. (2016) 49:14–23. doi: 10.1016/j.tifs.2016.01.003

[55] Alhakami AS Slovic P. A psychological study of the inverse relationship between perceived risk and perceived benefit. Risk Anal. (1994) 14:1085–96. doi: 10.1111/j.1539-6924.1994.tb00080.x, PMID: 7846317

[56] O’Connor EL White KM. Willingness to trial functional foods and vitamin supplements: the role of attitudes, subjective norms, and dread of risks. Food Qual Prefer. (2010) 21:75–81. doi: 10.1016/j.foodqual.2009.08.004

[57] Tzeng S-Y Ho T-Y. Exploring the effects of product knowledge, trust, and distrust in the health belief model to predict attitude toward dietary supplements. SAGE Open. (2022) 12. doi: 10.1177/21582440211068855

[58] Marsall M Engelmann G Skoda E-M Teufel M Bäuerle A. Validation and test of measurement invariance of the adapted health consciousness scale (HCS-G). Int J Environ Res Public Health. (2021) 18:6044. doi: 10.3390/ijerph18116044, PMID: 34199742 PMC8199981

[59] Marsall M Engelmann G Skoda E-M Teufel M Bäuerle A. Measuring electronic health literacy: development, validation, and test of measurement invariance of a revised German version of the eHealth literacy scale. J Med Internet Res. (2022) 24:e28252. doi: 10.2196/28252, PMID: 35107437 PMC8851340

[60] Bowman ND Goodboy AK. Evolving considerations and empirical approaches to construct validity in communication science. Ann Int Commun Assoc. (2020) 44:219–34. doi: 10.1080/23808985.2020.1792791, PMID: 40922668

[61] R Core Team. R: A language and environment for statistical computing (version 4.4.1). R Foundation for Statistical Computing (2024).

[62] Djaoudene O Romano A Bradai YD Zebiri F Ouchene A Yousfi Y. A global overview of dietary supplements: regulation, market trends, usage during the COVID-19 pandemic, and health effects. Nutrients. (2023) 15:3320. doi: 10.3390/nu15153320, PMID: 37571258 PMC10421343

[63] E W Stafford R MB. Health consciousness or familiarity with supplement advertising: what drives attitudes toward dietary supplements? Int J Pharm Healthc Mark. (2016) 10:130–47. doi: 10.1108/ijphm-06-2015-0026

[64] Pajor EM Oenema A Eggers SM de Vries H. Exploring beliefs about dietary supplement use: focus group discussions with Dutch adults. Public Health Nutr. (2017) 20:2694–705. doi: 10.1017/s1368980017001707, PMID: 28768564 PMC10261615

[65] Finucane M Alhakami AS Slovic P Johnson SM. The affect heuristic in judgments of risks and benefits. J Behav Decis Mak. (2000) 13:1–17. doi: 10.1002/(SICI)1099-0771(200001/03)13:1<1::AID-BDM333>3.0.CO;2-S

[66] Hatch AM Cole RE DiChiara AJ McGraw SM Merrill EP Wright AO. Personality traits and occupational demands are linked to dietary supplement use in soldiers: a cross-sectional study of sensation seeking behaviors. Mil Med. (2019) 184:e253–62. doi: 10.1093/milmed/usy201 30137399

[67] Dickinson A Blatman J El-Dash N Franco JC. Consumer usage and reasons for using dietary supplements: report of a series of surveys. J Am Coll Nutr. (2014) 33:176–82. doi: 10.1080/07315724.2013.875423, PMID: 24724775

[68] Conner M Kirk SFL Cade JE Barrett JH. Why do women use dietary supplements? The use of the theory of planned behaviour to explore beliefs about their use. Soc Sci Med. (2001) 52:621–33. doi: 10.1016/s0277-9536(00)00165-9, PMID: 11206658

[69] Colton JA Bower KM. Some misconceptions about R^2^. Int Soc Six Sigma Prof EXTRAOrdinary Sense. (2002) 3:20–2.

[70] German Federal Institute for Risk Assessment (BfR). Micronutrients and co. German Federal Institute for Risk Assessment (BfR). (2025). Available online at: https://www.microco.info/

[71] Garcia-Alvarez A Egan B de Klein S Dima L Maggi FM Isoniemi M. Usage of plant food supplements across six European countries: findings from the PlantLIBRA consumer survey. PLoS One. (2014) 9:e92265. doi: 10.1371/journal.pone.0092265, PMID: 24642692 PMC3958487

[72] O’Connor C Glatt D White L Revuelta Iniesta R. Knowledge, attitudes and perceptions towards vitamin D in a UK adult population: a cross-sectional study. Int J Environ Res Public Health. (2018) 15:2387. doi: 10.3390/ijerph15112387, PMID: 30373274 PMC6267199

