# Supplementary material for: Exploring motivations, information behavior, perceptions, and intentions among dietary supplement users: a cross-sectional survey study in Germany
Source: Front Nutr. 2025 Oct 7;12:1663562. doi: 10.3389/fnut.2025.1663562 (PMC12537374; doi:10.3389/fnut.2025.1663562)
Supplement: Supplementary file 1 [file Table_1.docx]

Supplementary Material

TABLE S1. Kinds of DS presented in the questionnaire*.*

| Category | Supplement | |
| --- | --- | --- |
| Vitamins (14) | (1) Vitamin A | (10) Beta-carotene (provitamin A) |
|  | (2) Vitamin B1 | (11) Folic acid (vitamin B9) |
|  | (3) Vitamin B2 | (12) Biotin (vitamin B7, vitamin H) |
|  | (4) Vitamin B6 | (13) Niacin (vitamin B3) |
|  | (5) Vitamin B12 | (14) Pantothenic acid (vitamin B5) |
|  | (6) Vitamin C |  |
|  | (7) Vitamin D |  |
|  | (8) Vitamin E |  |
|  | (9) Vitamin K |  |
| Minerals (17) | (1) Calcium | (10) Iodine |
|  | (2) Magnesium | (11) Fluoride |
|  | (3) Potassium | (12) Copper |
|  | (4) Iron | (13) Manganese |
|  | (5) Zinc | (14) Chromium |
|  | (6) Selenium | (15) Molybdenum |
|  | (7) Sodium | (16) Boron |
|  | (8) Chloride | (17) Silicon |
|  | (9) Phosphorus/phosphate |  |
| Botanicals (12) | (1) Bitter orange extract (synephrine) | (10) Milk thistle extract |
|  | (2) Aloe | (11) Ginkgo (ginkgo leaf extract) |
|  | (3) Curcumin | (12) Pumpkin seed extract |
|  | (4) Maca |  |
|  | (5) Valerian (valerian root extract) |  |
|  | (6) Hawthorn |  |
|  | (7) Spirulina |  |
|  | (8) Ashwagandha (sleeping berry) |  |
|  | (9) Ginseng (ginseng root extract) |  |
| Other substances (18) | (1) Omega fatty acids  (e.g., omega-3, fish oil, or algae oil) | (10) Red yeast rice |
|  | (2) Amino acids  (e.g., BCAA, leucine, lysine,  tryptophan, or glutamine) | (11) Chondroitin sulphate |
|  | (3) Proteins (e.g., whey protein) | (12) Coenzymes (e.g., Q10 or NADH) |
|  | (4) Probiotics or bacterial cultures  (e.g., lactic acid bacteria) | (13) Melatonin |
|  | (5) Prebiotics  (e.g., inulin, oligofructose, or lactulose) | (14) Creatine |
|  | (6) Hyaluronan | (15) Lactase |
|  | (7) Collagen | (16) Choline |
|  | (8) DMAA  (dimethylamylamine or methylhexanamine) | (17) CBD (cannabidiol) |
|  | (9) Glucosamine | (18) Spermidine |

Corresponding question: “Please indicate which of the following [vitamins | minerals | botanicals | other substances] you have taken in the previous 12 months via dietary supplements (e.g., as capsules or powder).”

TABLE S2. Item distributions for single-item measures.

|  |  | 1 (Very low) | | 2 | | 3 | | 4 | | 5 (Very high) | |  | |
| --- | --- | --- | --- | --- | --- | --- | --- | --- | --- | --- | --- | --- | --- |
|  | *n* | *n* | % | *n* | % | *n* | % | *n* | % | *n* | % | *M* | *SD* |
| Risk perception | 1,032 | 58 | 5.6 | 262 | 25.4 | 431 | 41.8 | 224 | 21.7 | 57 | 5.5 | 2.9 | 0.9 |
| Benefit perception | 1,035 | 94 | 9.1 | 202 | 19.5 | 446 | 41.1 | 253 | 24.4 | 40 | 3.9 | 2.9 | 0.9 |
|  |  | 1 (Very unlikely) | | 2 | | 3 | | 4 | | 5 (Very likely) | |  | |
|  | *n* | *n* | % | *n* | % | *n* | % | *n* | % | *n* | % | *M* | *SD* |
| Intentions to expand DS use | 1,055 | 323 | 30.6 | 297 | 28.2 | 170 | 16.1 | 186 | 17.6 | 79 | 7.5 | 2.4 | 1.3 |

*N* = 1,071.
